# Alkaline Protease Production from *Bacillus gibsonii* 6BS15-4 Using Dairy Effluent and Its Characterization as a Laundry Detergent Additive

**DOI:** 10.4014/jmb.2210.10007

**Published:** 2022-12-27

**Authors:** Polson Mahakhan, Patapee Apiso, Kannika Srisunthorn, Kanit Vichitphan, Sukanda Vichitphan, Sukrita Punyauppa-path, Jutaporn Sawaengkaew

**Affiliations:** 1 Department of Microbiology, Faculty of Science, Khon Kaen University, Khon Kaen 40002, Thailand; 2 Graduate School, Khon Kaen University, Khon Kaen 40002, Thailand; 3 Department of Biotechnology, Faculty of Technology, Khon Kaen University, Khon Kaen 40002, Thailand; 4 Fermentation Research Center for Value-Added Agricultural Products (FerVAAP), Khon Kaen University, Khon Kaen 40002, Thailand; 5 Department of Mathematics and Science, Faculty of Agriculture and Technology, Rajamangala University of Technology Isan Surin Campus, Surin 32000, Thailand

**Keywords:** Alkaline protease, isolation, dairy effluent, laundry detergent, *Bacillus gibsonii*

## Abstract

Protease is a widely used enzyme particularly in the detergent industry. In this research, we aimed to isolate alkaline protease-producing bacteria for characterization as a laundry detergent additive. The screening of alkaline protease production was investigated on basal medium agar plus 1% skim milk at pH 11, with incubation at 30°C. The highest alkaline protease-producing bacterium was 6BS15-4 strain, identified as *Bacillus gibsonii* by 16S rRNA gene sequencing. While the optimum pH was 12.0, the strain was stable at pH range 7.0-12.0 when incubated at 45°C for 60 min. The alkaline protease produced by *B. gibsonii* 6BS15-4 using dairy effluent was characterized. The optimum temperature was 60°C and the enzyme was stable at 55°C when incubated at pH 11.0 for 60 min. Metal ions K^+^, Mg^2+^, Cu^2+^, Na^+^, and Zn^2+^ exhibited a slightly stimulatory effect on enzyme activity. The enzyme retained over 80% of its activity in the presence of Ca^2+^, Ba^2+^, and Mn^2+^. Thiol reagent and ethylenediaminetetraacetic acid did not inhibit the enzyme activity, whereas phenylmethylsulfonyl fluoride significantly inhibited the protease activity. The alkaline protease from *B. gibsonii* 6BS15-4 demonstrated efficiency in blood stain removal and could therefore be used as a detergent additive, with potential for various other industrial applications.

## Introduction

Enzymes, acting as biological catalysts, are frequently utilized in many fields, including chemicals, fermentation, agriculture, pharmaceuticals, and food production. A global industrial enzymes market report covers the growing demand for glycohydrolases, proteases, lipases, polymerases, and nucleases for use in food and beverages, feed, bioethanol, detergents, pulp and paper, textiles and leather, and wastewater treatment. According to the recent research report by MarketsandMarkets, the industrial enzymes market was valued at US$ 10 billion in 2019 and is expected to reach US$ 14.7 billion by 2025, recording a compound annual growth rate (CAGR) of 6.7%, in terms of value [1]. Proteases or proteolytic enzymes are one of the most important groups of industrial enzymes accounting for more than 65% of the total market for industrial enzymes used in laundry detergent, brewing, the leather, dairy, pharmaceutical, marine and food industries, and in the production of protein. Proteases catalyze the digestion of proteins into short-chain peptides, or amino acids [2]. They are classified into two groups: (1) exopeptidases, which catalyze reaction at the ends of the peptide bonds from the C-or N-terminal; (2) and endopeptidases, which attack internal peptide bonds in the peptide chain. Endopeptidases are classified by their catalytic implementation into serine endoproteases (E.C. 3.4.21), cysteine endoproteases (E.C. 3.4.22), aspartic endoproteases (E.C. 3.4.23), metalloendoproteases (E.C. 3.4.24), and threonine endoproteases (E.C.3.4.25) [3].

Proteases are found in various organisms, such as plants, animals, and microorganisms. However, microbial proteases are preferred for production at the industrial level over plant and animal sources because microorganisms produce enzymes more easily and rapidly, and they are not influenced by climatic conditions or seasonal changes [4]. Moreover, microbial proteases are mostly found as extracellular enzymes, exhibiting rapid microbial growth on inexpensive media, easily produced on a large scale, economically feasible, with a high production yield whilst exhibiting high activity and stability at wide ranges of pHs and temperatures [5].

Alkaline proteases are a type of serine proteases, containing a serine and histidine as essential catalytic amino acid residues at their active sites, and catalyze hydrolytic reactions affecting amides and esters as well as peptides. Many alkaline proteases with high activity and stability in high alkaline range (pH 9-12) are interesting for numerous biotechnological and bioengineering applications [3]. They are a large group of enzymes, widely used in the detergent, food, pharmaceutical, and leather industries [6, 7]. In particular, alkaline proteases are an essential ingredient for the detergent industry. They are used as a supplementary ingredient in different brands of commercial detergents to increase cleaning efficiency by eliminating proteins such as milk, blood, and food stains. The action of alkaline proteases in a detergent depends on many factors such as pH, temperature, detergent composition, and the type of washing machine used. The key to utilizing alkaline protease as a complement in detergent commodities is the stability of the enzyme in the detergent. Therefore, we sought to identify a new type of alkaline protease with good, functional and stable properties that make it suitable as an additive in detergents.

Across several reports, alkaline proteases have been found in a number of different organisms; however, alkaline proteases from microorganisms in particular represent an attractive source for industrial enzymes. Various microorganisms such as fungi (*Aspergillus niger*, *A. fumigatus*, *A. reticulatus*, *Trametes cingulata*) [8-11], yeast (*Saccharomyces cerevisiae*, [12], *Aureobasidium pullulans*) [13], actinomycetes (*Streptomyces* sp. Al-Dhabi-82, *S. griseorubens*) [14, 15] and bacteria (*Bacillus licheniformis*, *B. alcalophilus*, *B. subtilis*, *B. alveayuensis*, *B. cereus*, *Pseudoalteromonas arctica*) [16-20] are known to produce these enzymes.

Even though numerous microorganisms are identified as producing alkaline proteases, *Bacillus* spp. are the preferred main sources for commercial and industrial enzymes owing to their exceptional ability to secrete large amounts of highly active enzymes, whilst some of these genera are generally regarded as safe. The genus *Bacillus*, belonging to the family *Bacillaceae*, is gram-positive, rod-shaped, and endospore-forming [21]. In addition, *Bacillus* plays a particularly role in industry; it is the best producer of alkaline protease with pH activity values above 7.0, while approximately half of all commercial protease production is obtained from *Bacillus* spp. [22].

Therefore, the isolation and screening of new, alkaline protease-producing *Bacillus* strains from natural habitats, could pave the way for new opportunities and developments. The isolated strains could be used for observing and producing novel alkaline protease in various industries and in highly alkaline conditions. This research aimed to screen, isolate, identify, and characterize the excellent, alkaline protease-producing bacteria collected from the Resources Protection Area of Khok Phutaka, Khon Kaen Province, under the Plant Genetics Conservation Project under the Royal Initiation of her Royal Highness Princess Maha Chakri Sirindhorn. In addition, dairy effluent was used as a raw material to produce alkaline protease from selected bacteria because it is cheap and rich in nutrients. Finally, the efficiency of the crude enzyme as a detergent additive was also evaluated.

## Materials and Methods

### Isolation of Alkaline Protease-Producing Bacteria

Soil samples collected from the Resources Protection Area in Khok Phutaka, Khon Kaen Province under the Plant Genetics Conservation Project under the Royal Initiation of her Royal Highness Princess Maha Chakri Sirindhorn were diluted and homogenized in 0.9% sterile saline solution. The suspension was streaked on basal medium plus 1% skim milk agar plate (BMSM: 1.0% glucose, 1% skim milk, 0.5% yeast extract, 0.1% K_2_HPO_4_, 0.02% MgSO_4_.7H_2_O, 1.0% Na_2_CO_3_, agar 1.5%, pH 11) [23] and incubated at 30°C for 72 h. The alkaline protease production bacteria were observed for protein hydrolysis by formation of clear zones around the colonies. The diameters of the bacterial colonies (CO) and the clear zones around the bacterial colonies (CZ) were measured. The difference between CZ and CO (CZ - CO) and the ratio between CZ and CO (CZ / CO) were compared. The selected strains with maximum high values of both calculations were further analyzed for alkaline protease activities.

### Alkaline Protease Production and Enzyme Assay

The selected strains were cultured in BMSM, pH 11, and incubated at 30°C with shaking at 120 rpm for 0-192 h. Samples were collected every 24 h to analyze cellular growth and alkaline protease activity. The cell culture was centrifuged (10,000 ×*g*, 10 min) and the supernatant was recovered as crude protease for analysis. The cell sediment was determined for cellular growth by viable plate count.

Alkaline protease assay was measured by the modified method using casein as a substrate [24]. The mixture, a diluted enzyme 1 ml and 1 ml of 1% casein (in glycine-NaOH buffer, pH 11.0) was incubated at 45°C for 15 min followed by the addition of 2 ml of 10% trichloroacetic acid (TCA). It was then left at 4°C for 1 h to allow complete or almost complete precipitation of protein. The control sample was also prepared by adding TCA before the enzyme addition. The reaction mixture was centrifuged, and the supernatant was measured for tyrosine concentration [25]. One unit of enzyme alkaline protease was defined as the amount of protease that released 1 μg/ml of tyrosine from casein per minute under experiment conditions.

### Identification of Alkaline Protease-Producing Bacteria

Molecular identification of the selected strain was achieved by sequence analysis of 16S rRNA gene. The 16S rRNA gene was amplified using forward primer 785F (5′-GGA TTA GAT ACC CTG GTA-3′) and reverse primer 907R (5′-CCG TCA ATT CMT TTR AGT TT) 3′). The PCR products were purified and sequenced by Macrogen Inc., Korea. The sequence was submitted, and the accession number was provided by GenBank.

### Preparation of Inoculum and Alkaline Protease Production on Dairy Effluent

Alkaline protease production was carried out in two steps. First, the selected strain was cultured in BMSM, pH 11, and incubated at 30°C with shaking at 120 ×*g* for 24 h. Second, the seed culture was inoculated (1%v/v) into 100 ml of dairy effluent supplement with basal medium (1.0% glucose, 0.5% yeast extract, 0.1% K_2_HPO_4_, 0.02%MgSO_4_.7H_2_O, 1.0% Na_2_CO_3_) plus 0, 0.25, or 0.5% skim milk, and the pH was adjusted to 11 before incubation at 30°C with shaking at 120 rpm for 168 h. The enzyme was collected for alkaline protease characterization and compared with basal medium plus 1% skim milk (conventional media) cultivation.

### Effect of pH and Temperature on Enzyme Activity and Stability

Alkaline protease activity was measured at a pH range of 3 to 13 at 45°C for 15 min using casein as a substrate. The pH stability was determined by preincubation in various buffer solutions with different pH values for 60 min at 45°C. The enzymes were withdrawn, and remaining enzymatic activities were determined at pH 11 and 45°C. The four buffers system used in this study consisted of 0.05 M acetate buffer (pH 3-6), 0.05 M phosphate buffer (pH 6-8), 0.05 M glycine-NaOH buffer (pH 8-11), and 0.05 M hydroxide-chloride buffer (pH 11-13).

The optimum temperature of enzyme activity was assessed at between 30 and 80°C using casein as a substrate in 0.05 M glycine-NaOH buffer, pH 11. Temperature stability was investigated by incubating the enzyme in 0.05 M glycine-NaOH buffer, pH 11.0, for 60 min over a temperature range of 30 to 80°C. The enzymes were withdrawn, and remaining enzymatic activities were determined at pH 11 and 45°C.

### Effect of Metal Ions, Surfactants, Oxidizing Agents and Enzyme Inhibitors

The effects of 8 metal ions (5 mM; Na^+^, Ca^2+^, K^+^, Mn^2+^, Zn^2+^, Cu^2+^, Ba^2+^, Mg^2+^) were investigated by addition to the reaction mixture. The enzyme assay was carried out as described earlier. The effect of various surfactants (1%(v/v) Tween 80, 0.1% (w/v) SDS, oxidizing agents (0.1% (v/v) hydrogen peroxide, 1.5% (v/v) sodium perborate and protease inhibitors (5 mM 5,5'-dithiobis-(2-nitrobenzoic acid) (DTNB), 5 mM phenylmethylsulfonyl fluoride (PMSF), 5 mM ethylenediaminetetraacetic acid (EDTA), 5 mM β-mercaptoethanol (BME)) on alkaline protease activity were investigated by adding those chemicals to the reaction mixture. Relative enzyme activities were determined. The alkaline protease activity obtained without inhibitor was considered as 100% relative activity.

### Washing Performance Test with Alkaline Protease

The efficiency of alkaline protease (0.316 units) as a detergent additive was studied on white cotton cloth pieces (5 × 5 cm) stained with bovine blood. Fresh bovine blood (100 μl) was spread on the surface of each piece of white cotton cloth, and the pieces were left in the oven (60°C) for 4 h to dry and fix the stain. The following set experiments were prepared and tested with (a) 200 μl distilled water, (b) 200 μl glycine-NaOH buffer, pH 11.0, (c) 100 μl crude enzyme plus 100 μl detergent, (d) 100 μl detergent solution100 μl glycine-NaOH buffer, pH 11.0, and (e)100 μl crude enzyme plus 100 μl glycine-NaOH buffer, pH 11.0. Each condition was dropped on the bovine blood-stained cotton pieces and incubated at 45°C for 60 min. After incubation, each piece of cloth was washed with tap water for 2 min and air-dried for 4 h. Untreated bovine blood-stained cotton pieces with enzyme were taken as control. The blood stain removal was determined by visualization.

### Hydrolysis of Protein Substrates

The ability of alkaline protease enzyme to hydrolyze blood, eggs, chicken feathers and milk were investigated. The samples were mixed with alkaline protease enzyme (158.44 units). The mixtures were incubated at 30°C for 3 days and samples were taken every day to determine the protein hydrolysis ability. The amino acid from the protein solution for each hydrolysis reaction was determined by using the Lowry method.

## Results

### Isolation of Alkaline Protease-Producing Bacteria

**Fig. 1:**
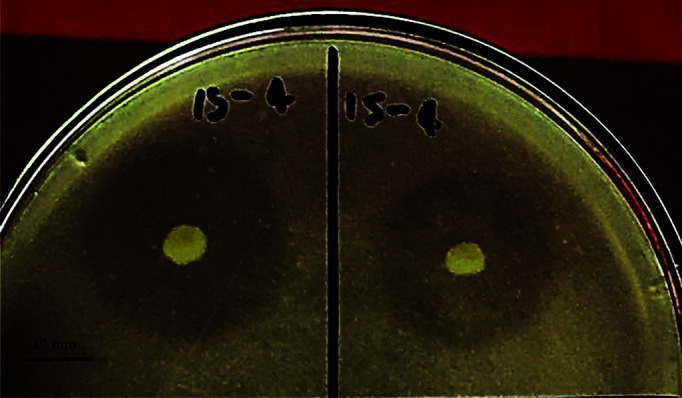
Alkaline protease-producing bacteria *B. gibsonii* 6BS15-4 on BMSM agar plates showing zone of proteolysis when incubated at 30°C for 72 h (scale bar = 10 mm).

Few cultures appeared owing to the high alkaline condition of the isolation media. The alkaline protease production bacteria were observed for protein hydrolysis by formation of clear zones around the colonies. Photographs of protein hydrolyzed zones are presented in Fig. 1. The 169 isolates showing a clear zone of proteolysis were point inoculated on BMSM agar for calculating the difference between CZ and CO (CZ - CO) and the ratio between CZ and CO (CZ / CO). Among those isolates, the 13 isolates which obtained high values of both (CZ - CO) and (CZ / CO) ratio were selected for further alkaline protease determination. Bacterial isolate 6BS15-4 showed the highest difference between CZ (27.5 mm) and CO (6.3 mm) and the greatest ratio between CZ and CO, at 21.2 mm and 4.37, respectively.

**Fig. 2:**
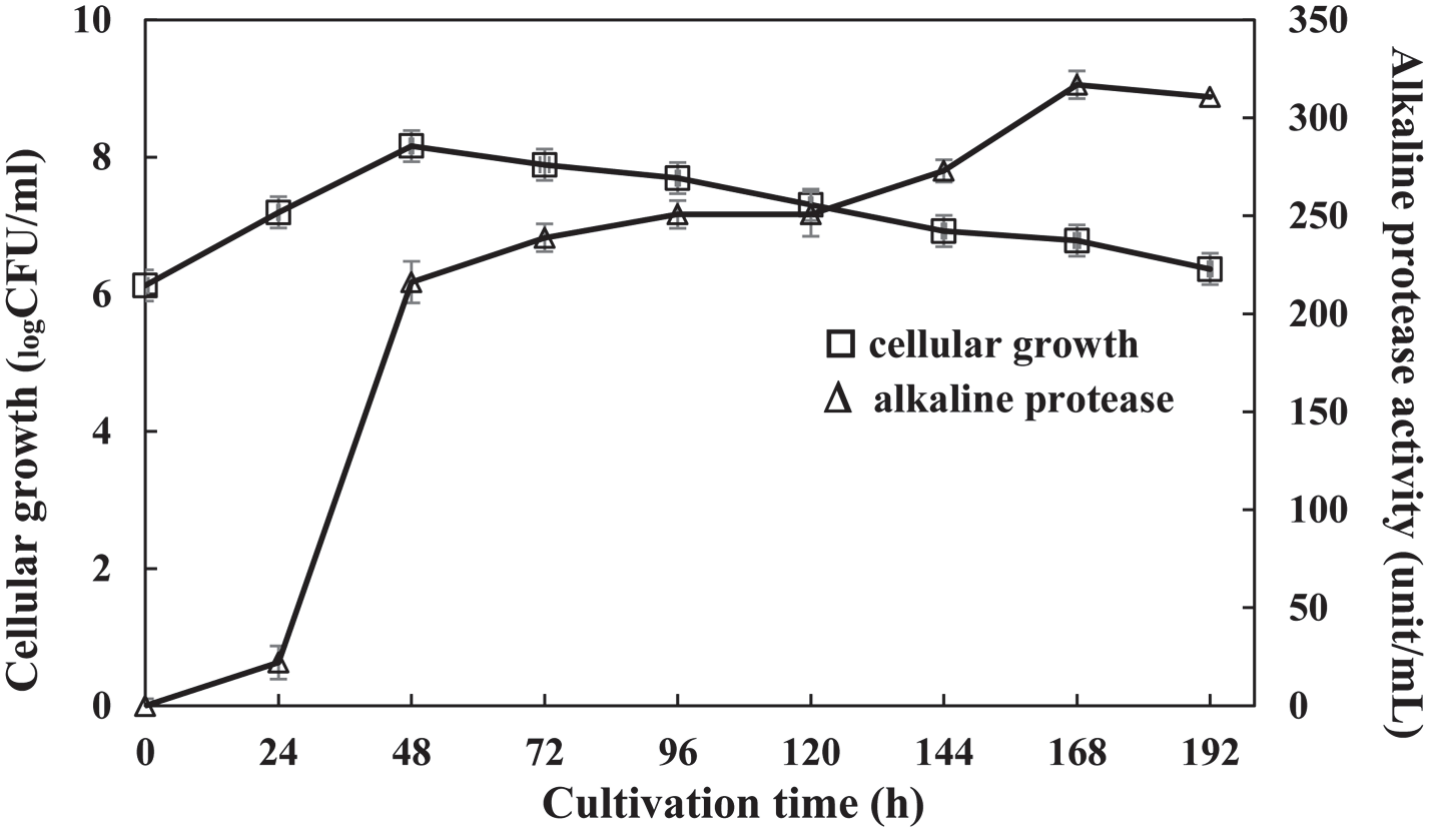
Time course of cellular growth (-□-) and alkaline protease production (-△-) of *B. gibsonii* 6BS15-4 when cultured in BMSM, pH 11, and incubated at 30°C with shaking at 120 rpm for 192 h.

The 13 isolates were further screened quantitatively for the production of alkaline protease in BMSM broth with the shake flask. The highest alkaline protease-producing bacterium was 6BS15-4 strain (316.88 units/ml) within 168 h of cultivation. Alkaline protease production started after a 24-h lag phase, then increased exponentially along with alkaline protease production from 24-48 h. From stationary phase to dead phase, cellular growth and enzyme production were exhibited in the range 48-192 h of cultivation. Time course of the cellular growth and alkaline protease production of 6BS15-4 strain are shown in Fig. 2. In addition, 6BS15-4 strain was identified as *Bacillus gibsonii* by 16S rRNA gene sequencing. The results revealed that strain 6BS15-4 exhibited a high level of 16S rRNA gene similarity (99%) with *B. gibsonii* NR_026143.1.

### Alkaline Protease Production on Dairy Effluent

Dairy effluent supplement was collected from a dairy cow farm. The total protein and reducing sugar were observed at 766.60 mg/l and 381.30 mg/l, respectively. Relative activity of alkaline protease production from *B. gibsonii* 6BS15-4 when cultured in various culture media compared with conventional medium are shown in Table 1.

Alkaline protease activity production from *B. gibsonii* 6BS15-4 using dairy effluent supplement with basal medium obtained 72.95% relative activity of alkaline protease activity when compared with alkaline protein cultivation from conventional media. Moreover, enzyme production had few differences when cultured in dairy effluent supplement with basal medium plus 0, 0.25, or 0.5% skim milk. Therefore, dairy effluent supplement with basal medium could be used for enzyme production.

### Effect of pH and Temperature on Enzyme Activity and Stability

**Fig. 3:**
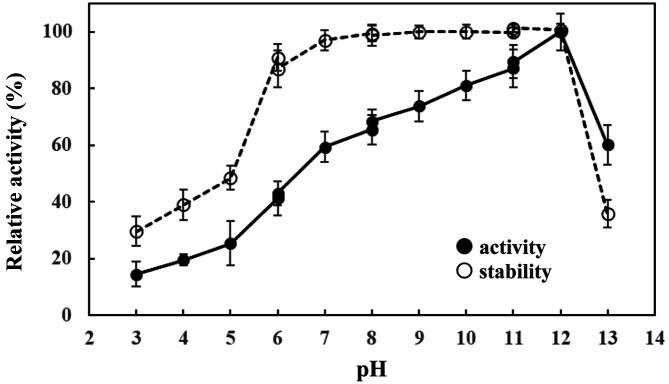
The effects of various pH on the alkaline protease activity (-●-) and stability (-○-) of *B. gibsonii* 6BS15-4.

The appropriate enzymes for laundry detergent additives should have both high activity and high stability over a broad range of pH and temperatures. The pH activity profiles of the alkaline protease of *B. gibsonii* 6BS15-4 were determined with various pH ranges from 3 to 13. The result showed maximum activity at pH 12 and retention of around 80% of relative activity over a pH range of 10-12, while the enzyme activity was observed at around 50% of relative activity at pH 13. The results of pH stability revealed that this enzyme was 100% stable even at pH 12 for 60 min. It retained more than 85% of relative activity over a pH range of 6 to 12 when incubated at 45°C for 60 min (Fig. 3).

**Fig. 4:**
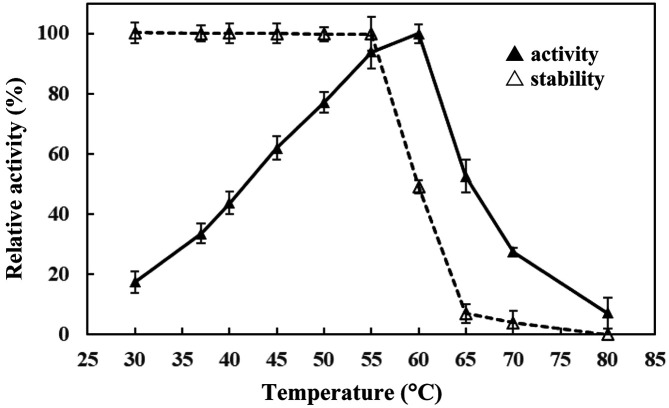
The effects of various temperatures on the alkaline protease activity (-▲-) and stability (-△-) of *B. gibsonii* 6BS15-4.

Temperature activity profiles of the alkaline protease of *B. gibsonii* 6BS15-4 were determined with temperature ranges of 30-80°C. The effect of temperatures on alkaline protease enzyme activity was observed and the results are shown in Fig. 4. Alkaline protease enzyme activity was highest in the range of 55-60°C, with an optimum temperature of 60°C for 60 min, but rapidly decreased at temperatures higher than 60°C, while enzyme activity was observed at around 50% of relative activity at 65°C. The alkaline protease of *B. gibsonii* 6BS15-4 was stable at 30-55°C for 60 min of incubation, pH 11. However, while the activity was rapidly lost above 55°C, around 50% of the relative activity was retained at 60°C.

The buffers were 0.05 M acetate buffer (pH 3-6), 0.05 M phosphate buffer (pH 6-8), 0.05 M glycine-NaOH buffer (pH 8-11), and 0.05 M hydroxide-chloride buffer (pH 11-13).

### Effect of Metal Ions, Surfactants, Oxidizing Agents, and Enzyme Inhibitors

The effects of 8 metal ions (final concentration of 5mM; Na^+^, Ca^2+^, K^+^, Mn^2+^, Zn^2+^, Cu^2+^, Ba^2+^, Mg^2+^) on alkaline protease from *B. gibsonii* 6BS15-4 are shown in Table 3. Metal ions testing showed a slightly stimulatory effect of K^+^, Mg^2+^, Cu^2+^, Na^+^ and Zn^2+^ on enzyme activity of around 118, 114, 114, 108 and 110%, respectively. The enzyme retained 84% of its activity in the presence of Mn^2+^, while enzyme activities were observed at around 91% and 97%in the presence of Ba^2+^ and Ca^2+^, respectively.

The alkaline protease of *B. gibsonii* 6BS15-4 activity was stable in the presence of 1% (v/v) Tween 80, 0.1% (v/v) hydrogen peroxide, and 1.5% (v/v) sodium perborate. In contrast, the enzyme activity decreased by 21.5% in the presence 0.1% (w/v) SDS. Thiol reagent and ethylenediaminetetraacetic acid did not inhibit enzyme activity, whereas phenylmethylsulfonyl fluoride did so significantly. Therefore, this enzyme could likely be a serine-type protease (Table 4).

### Washing Performance Test with Alkaline Protease

**Fig. 5:**
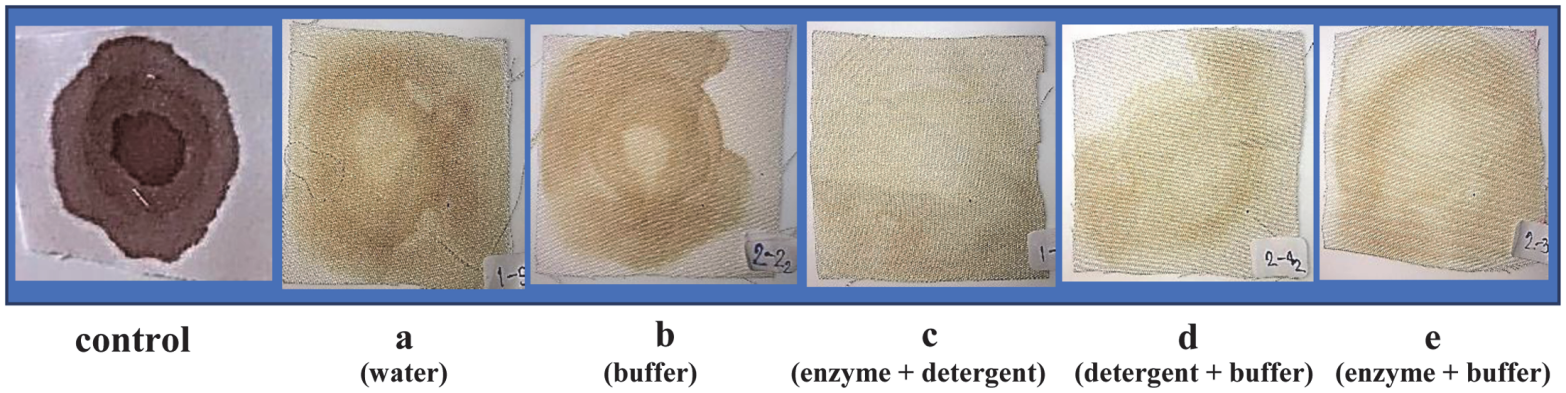
Application of alkaline protease of *B. gibsonii* 6BS15-4 on blood stain removal.

The different treatment conditions of crude enzyme from *B. gibsonii* 6BS15-4 on blood stain removal are shown in Fig. 5. The alkaline protease of *B. gibsonii* 6BS15-4 plus detergent appeared to improve the cleansing process as evidenced by rapid blood stain removal from cotton cloth pieces when compared to enzyme or detergent alone.

The set including (a) 200 μl distilled water, (b) 200 μl glycine-NaOH buffer pH 11.0, (c) 100 μl crude enzyme + 100 μl detergent (d) 100 μl detergent solution 100 μl glycine-NaOH buffer pH 11.0, (e) 100 μl crude enzyme + 100 μl glycine-NaOH buffer pH 11.0 was dropped on the bovine blood-stained cotton pieces and incubated at 45°C. for 60 min. Untreated bovine blood-stained cotton pieces with enzyme were taken as control.

### Hydrolysis of Protein Substrates

**Fig. 6:**
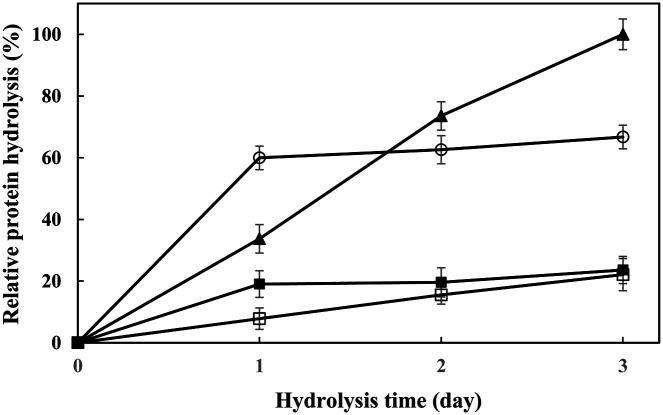
Relative protein hydrolysis of blood (-■-), egg solution (-▲-), chicken feathers (-□-) and milk (-○-) by alkaline protease of *B. gibsonii* 6BS15-4 at 30°C for 3 days.

The ability of alkaline protease to hydrolyze blood, egg solution, chicken feathers and milk was investigated at 30°C for 3 days. At day 1 of incubation, milk yielded the highest protein hydrolysis, chicken feathers the lowest. However, alkaline protease of *B. gibsonii* 6BS15-4 showed the highest capacity for hydrolysis of egg solution at 3 days of incubation (Fig. 6).

## Discussion

The isolation and screening of new, alkaline protease-producing bacteria from an abundance of natural resources may reveal new opportunities to observe some novel alkaline protease-producing bacteria that could be used in highly alkaline conditions in various industries. The highest alkaline protease-producing bacterium isolated in this study was *B. gibsonii* 6BS15-4. Proteases produced by *Bacillus* species are the most important group of protease enzymes currently being industrially exploited. The results presented here agree with the literature, as several *Bacillus* species are known to be good alkaline protease producers and have been widely used in various industries [15-19].

The study on growth pattern and alkaline protease production of *B. gibsonii* 6BS15-4 was carried out in a shake flask containing 100 ml of BMSM at pH 11 and incubated at 30°C with shaking at 120 rpm for 192 h. The maximum proteolytic activity (316.88 units/ml) was observed after 168 h of growth where it reached stationary phase. *B. gibsonii* 6BS15-4 grew rapidly but scarcely produced alkaline protease in early log phase. However, in log phase (24-48 h), the protease increased substantially, and the maximum proteolytic activity (316.88 units/ml) was observed after 168 h of growth where it reached dead phase. The growth cycle of this bacteria has a very short stationary phase, possibly due to a stress environment condition or limited nutrients. The enzymes were found to be highly productive during the dead phase, possibly due to limited nutrients. According to the above results, it could be assumed that alkaline protease production was partially associated with microbial cell growth. Similar dynamics of protease production from *B. circulans* [29] and *B. subtilis* [30] have also been reported. The enzyme activity of *B. firmus* MTCC7728 was studied in relation to growth and the maximum reached at stationary phase was found to be 215 units/ml [31].

Food industries are notorious for high water consumption and production of enormous amounts of effluents, sludge, and waste. A dairy effluent supplement collected from cow dairy effluent was used in this study. Milk is comprised of nutritive compounds such as caseins and whey proteins. Caseins constitute 80% of the total protein present in milk [32]. Therefore, microorganisms are considered sources of cellular growth and protease production. Alkaline protease activity production from *B. gibsonii* 6BS15-4 using dairy effluent supplement with basal medium obtained 72.95% of alkaline protease activity when compared with alkaline protein cultivation from conventional media.

The dairy effluent supplement with basal medium with no added skim milk could save 38% of costs. However, enzyme productivity was 73%, thus the total saving was 15% when compared with the conventional media. The use of dairy effluent to produce alkaline protease is the eco-friendly and economical method for bioconversion of effluent wastes into value-added products, in addition, environmental pollution could be decreased. Moreover, the low cost of enzyme production eventually leads to a decrease in production cost.

Protease with the unique properties of pH stability and thermostability towards solvents, oxidizing agents, and surfactants are of particular use in industry. Alkaline proteases generally have optimal activities over a broad pH range of pH 5 to 11 and stability of protease enzyme in a broad pH range of pH 6 to 11 in *B. gibsonii* aprBG [28], *B. licheniformis* MH31 [33] and *B. licheniformis* Bl8 [34]. Meanwhile, in this study the enzyme showed maximum activity at pH 12 and retained activity over a pH range of 6 to 12, higher than in other reports. This confirmed the promising potential of alkaline protease from *B. gibsonii* 6BS15-4 for future industrial application, which requires enzyme stability at a wide pH range. Furthermore, the protease was active over a broad range of temperatures and had an optimum activity at 60°C, while other alkaline proteases of *Bacillus* spp. with optimal temperature at 50°C have also been reported [34]. The thermostability investigations of the alkaline protease from *B. gibsonii* 6BS15-4 indicated that the protease was absolutely stable below 55°C for 60 min incubation and pH 11. However, an optimum temperature of 50°C has been reported in *B. gibsonii* aprBG [28] and *B. licheniformis* Bl8 [34].

The enzyme showed maximum activity at pH 12 and retained activity over a pH range of 6 to 12 (Fig. 2). Meanwhile, *B. gibsonii* aprBG was also observed from protease over a range of pH from 5 to 11, with optimal activity at pH 9.5 [28].

None of the metal ions showed a considerable enhancing effect on the activity of the alkaline protease. However, K^+^, Cu^2+^ and Mg^2+^ have demonstrated enhancing effects on the activity of the reported alkaline proteases from some other bacterial sources, such as *B. alveayuensis* CAS 5 [18], *B. subtilis*SD11 [35], and *B. safensis* strain RH12 [36]. Here, the surfactants and oxidizing agents showed no effect on *B. gibsonii* 6BS15-4 alkaline protease activity. Thus, alkaline protease in this study was significantly more stable in the presence of non-ionic, anionic, and commercial detergents, which would make this enzyme a viable detergent additive. Additionally, the results of the blood stain removal experiment showed complete stain removal in detergent solution supplemented with enzyme, whereas the blood stain was not completely removed by the detergent solution alone.

Considering the production costs and reutilization of bioresources, alkaline protease production by microbial action seems to be promising approach; the use of dairy wastewater to produce protease is an inexpensive and eco-friendly method for the bioconversion of these wastes into value-added products and, for reducing environmental pollution. The alkaline protease obtained from *B. gibsonii* 6BS15-4 has high stability towards temperature and pH, and is also stable in the presence of non-ionic, anionic, and commercial detergents. These properties indicate its possible use in the commercial manufacture of detergent. Therefore, the alkaline protease in our study is proposed as an additive for detergents to enhance washing performance.

## Figures and Tables

**Table 1:** Relative alkaline protease activity production from *B. gibsonii* 6BS15-4 when cultured in various culture media, pH 11, and incubated at 30°C with shaking at 120 ×*g* for 168 h.

Cultivation media	Relative activity (%)
Basal medium + 1% skim milk (conventional media)	100 ± 7.89
Dairy effluent + basal medium + 0.25% skim milk	78.85 ± 8.87
Dairy effluent + basal medium + 0.5% skim milk	76.52 ± 3.92
Dairy effluent + basal medium + 0% skim milk	72.95 ± 1.71

**Table 3:** The effect of various metal ions on *B. gibsonii* 6BS15-4 alkaline protease activity.

Metal ions (5 mM)	Relative activity (%)
Control	100.00 ± 2.18
Na^+^ (NaCl)	108.11 ± 1.82
Ca^2+^ (CaCl_2_)	96.51 ± 2.02
K^+^ (KCl)	117.95 ± 2.52
Mn^2+^ (MnCl_2_)	83.76 ± 1.28
Zn^2+^ (ZnCl_2_)	109.68 ± 2.71
Cu^2+^ (CuSO_4_)	114.52 ± 2.04
Ba^2+^ (BaCl_2_)	90.77 ± 2.66
Mg^2+^ (MgSO_4_)	114.31 ± 2.06

Alkaline protease activity measured in the absence of any metal ions was reserved as control (100%). The enzyme activity was measured at pH 11 and 45°C.

**Table 4:** The effect of surfactants and oxidizing agent sand enzyme inhibitors on *B. gibsonii* 6BS15-4 alkaline protease activity.

Chemical	Relative activity (%)
Control	100.00 ± 1.13
Surfactants	
1% (v/v) Tween 80	99.59 ± 2.69
0.1% (w/v) SDS	78.57 ± 2.03
Oxidizing agents	
0.1% (v/v) hydrogen peroxide	104.32 ± 1.70
1.5% (v/v) sodium perborate	108.00 ± 0.66
Enzyme inhibitors	
5 mM 5,5'-dithiobis-(2-nitrobenzoic acid) (DTNB)	95.98 ± 4.06
5 mM Phenylmethylsulfonyl fluoride (PMSF)	28.90 ± 0.22
5 mM thylenediaminetetraacetic acid (EDTA)	99.94 ± 0.22
5 mM β-mercaptoethanol (BME)	102.85 ± 0.17

Alkaline protease activity measured in the absence of any chemical agent was reserved as control (100%). Enzyme activity was measured at pH 11 and 45°C.
